# Supplementary material for: Contrastive learning enhanced retrieval-augmented few-shot framework for multi-label patent classification
Source: PLoS One. 2026 Jan 21;21(1):e0341118. doi: 10.1371/journal.pone.0341118 (PMC12822942; doi:10.1371/journal.pone.0341118)
Supplement: Attachment — Submitted filename: review_response.pdf [file pone.0341118.s002.pdf]

We would like to sincerely thank the reviewers for their helpful remarks and corrections. Our response to the raised points can be found below. We hope that the revised manuscript addresses all issues in a satisfying manner; all changes made to the article are marked with blue highlighting.

Reviewer #1 comments

1. " *The evaluation is confined to a single domain with a custom 10-category schema. Results may not transfer to other technical domains. Evaluating on other domains would strengthen claims of generality.* "

    We sincerely thank the reviewer for this important observation. We acknowledge that our evaluation is confined to UAV patent classification, and we agree that broader evaluation would strengthen generalizability claims. We selected the UAV domain deliberately as it exemplifies multi-label classification challenges through its inherent multidisciplinary nature, encompassing mechanical, electronic, software, and communication technologies within individual patents. This characteristic makes it an ideal testbed for validating our approach under realistic multi-label conditions.

    In response to this valuable feedback, we have added a comprehensive limitations paragraph in the Discussion section that explicitly acknowledges the domain-specific evaluation and discusses the need for additional validation across other technological domains. The limitations discussion addresses (1) the need for cross-domain validation beyond UAV patents, (2) computational costs and API dependencies from GPT-4o integration, (3) domain-specific data requirements for contrastive pre-training, and (4) challenges with non-English patents and genuinely novel technologies. We also outline future research directions including evaluating across multiple patent categories, exploring lightweight LLM alternatives, and investigating transfer learning strategies to reduce domain-specific data requirements.

    While our current focus remains on demonstrating the framework's effectiveness within the patent domain, we believe the modular architecture and methodological contributions provide a foundation for future cross-domain evaluation.

2. " *Efficiency is under-reported. The paper should provide efficiency analysis like a parameter/GFLOPs comparison to baselines.* "

    We thank the reviewer for highlighting this important aspect. We have added a dedicated "Computational efficiency" subsection in the Results section with comprehensive efficiency analysis. Table 4 presents detailed comparisons including parameters, GFLOPs, inference time, GPU memory, and training time across all eight baseline methods, organized into patent-specific methods (LLM-AL, PatentSBERTa) and general baselines (RoBERTa-Large, XLNet-Large, RePrompt, RAG+BERT, Prototypical, META-LSTM).

3. "*The figure is very vague. The authors need to refine the resolution of the figures to make it readable.*"

We appreciate this feedback. Following PLOS ONE submission guidelines, figures in the LaTeX manuscript are placeholders, as the template requires figures to be uploaded separately. We have provided all figures as high-resolution (600 DPI) files in the revised submission package to ensure optimal readability.

4. “

   Missing related work. Several recent works relevant to few-shot learning are relevant and should be cited:

   (a) *Multimodality Helps Few-shot 3D Point Cloud Semantic Segmentation (ICLR 2025)*
   (b) *Generalized Few-shot 3D Point Cloud Segmentation with Vision-Language Model (CVPR 2025)*
   (c) *In-Context Learning for Text Classification with Many Labels (ACL 2023)*

   ”

   We thank the reviewer for these valuable suggestions. We have incorporated all three recommended citations into the ”Retrieval-augmented few-shot learning” subsection. Specifically, we cite Milios et al. (2023) on in-context learning for multi-label text classification, which directly relates to our demonstration selection approach. We also cite An et al. (2024, 2025) on few-shot 3D point cloud segmentation to acknowledge recent advances in few-shot learning across computer vision domains, noting that cross-domain insights may inform future methodological developments.

Reviewer #2 comments

1. “ *The ”Background and related work” section covers too many research directions (six in total) and employs an overly complex classification scheme. It is recommended to streamline and consolidate this part for better clarity and focus.* ”

   We appreciate this constructive feedback. We have streamlined the Background and related work section from six subsections to four by consolidating related content. Specifically, we merged ”Multi-label patent classification,” ”Domain-specific pre-training and technical language models,” and ”UAV patent classification” into a single unified subsection titled ”Patent classification with domain-specific language models.” This consolidation integrates patent-specific challenges, domain adaptation strategies, and UAV testbed justification into a coherent narrative. The revised structure maintains focus on the four core methodological pillars: patent-specific models, contrastive learning, retrieval-augmented few-shot learning, and chain-of-thought reasoning.

2. “ *The ”Dataset” subsection should clarify whether the dataset is a novel contribution of this work or was obtained from an existing source, providing appropriate citations in either case.* ”

   We thank the reviewer for this important clarification request. We have explicitly stated in the Dataset subsection that the curated UAV patent dataset with 15,000 expert-annotated patents across ten technological categories represents a novel contribution of this work. We have also added a footnote with the GitHub repository URL for dataset access and citation.

3. “ *Figures should be embedded close to their corresponding references in the main text rather than being placed later in the document. Additionally, the current image resolution is insufficient and should be improved.* ”

   We appreciate this suggestion. Following PLOS ONE submission guidelines, we have repositioned all figures to appear immediately after their first citation in the text. All figures have been provided as high-resolution (600 DPI) files in the revised submission package.

4. “*For Table 3, please include citations for the comparative algorithms listed. Furthermore, the note stating "All improvements by our framework are statistically significant ($p <$ 0.001)" should be supported with relevant details in the text explaining where and how statistical significance was evaluated, as well as what this implies for the results.*”

   We thank the reviewer for this important observation. We have added citations for all baseline algorithms in Table 3 and substantially expanded the statistical significance explanation in both the revised manuscript and table footnote to include detailed methodology, multiple comparison correction, and effect sizes.

5. “*It is recommended to present the ablation study results in a tabular format, which would more clearly demonstrate the contribution of each individual component.*”

   We appreciate this suggestion. We have presented the ablation study results in a tabular format (now Table 5) in the "Ablation study" subsection.

6. “*Among the six algorithms currently included in "Overall performance comparison" section, it is necessary to supplement with 2–3 recently proposed algorithms specifically designed for patent classification.*”

   We appreciate this valuable suggestion. We have supplemented the comparison with two recently proposed patent-specific methods: (1) LLM-AL (Xiong et al., 2025), an iterative large language model with active learning, and (2) PatentSBERTa (Bekamiri et al., 2024), a hybrid SBERT-KNN model with patent-specific domain adaptation. We reorganized Table 3 to distinguish recent patent-specific methods from general baselines, updated statistical tests for eight comparisons, and added analysis demonstrating our framework's improvements over these state-of-the-art patent classification approaches.

7. “*The references contain formatting inconsistencies. Journal and conference names should follow standard capitalization rules (e.g., each major word capitalized). Additionally, page numbers are missing in several entries and must be provided.*”

   We thank the reviewer for this careful observation. We have corrected all formatting inconsistencies in the bibliography: (1) Capitalized journal names following standard rules ("arXiv Preprint," "IEEE Access," "Advances in Neural Information Processing Systems"), (2) For entries lacking page numbers, we have either added them where available or appropriately classified entries as working papers or theses where page numbers do not apply. All journal articles now include complete bibliographic information.

Again, we are grateful to the reviewers for their time and effort, and we sincerely thank them for their valuable help in improving our manuscript.

Best regards, on behalf of the authors,
Shikun Chen
